# Improving the Acute Management of Ureteric Stones in a District General Hospital: A Two-Phase Quality Improvement Study Evaluating Practice Against NICE NG118 and GIRFT Recommendations

**DOI:** 10.7759/cureus.89902

**Published:** 2025-08-12

**Authors:** Asmita Hossain, Khaldoun Fozo, Panagiotis Papikinos

**Affiliations:** 1 Urology, Surrey and Sussex Healthcare NHS Trust, Redhill, GBR

**Keywords:** eswl, girft, medical expulsion therapy (met), mimic score, nice ng118, nsaid, primary ureteroscopy, ureteric colic, ureteric jj stent, ureteric stones

## Abstract

Background: Ureteric colic is one of the most common urological emergencies. Adherence to the National Institute for Health and Care Excellence (NICE) guideline NG118 and the Getting It Right First Time (GIRFT) recommendations can improve care delivery by reducing unnecessary stenting and facilitating timely definitive management. However, implementation in district general hospitals may vary due to logistical constraints.

Objective: This study aims to evaluate current compliance with NICE NG118 and GIRFT standards in the acute management of ureteric stones at a UK district general hospital and to assess the impact of targeted interventions on adherence to these guidelines between two audit cycles.

Methods: A retrospective two-phase audit was conducted: Phase 1 (Aug-Nov 2023) and Phase 2 (Jul-Sep 2024). Data were collected on adults admitted with ureteric stones, including demographics, imaging, analgesia, treatment modality, stent use, and follow-up. Interventions between phases included creating a primary ureteroscopy (URS) and day-case extracorporeal shock wave lithotripsy (ESWL) pathway, an electronic stent recall protocol, Cerner auto-text discharge summaries, departmental poster and oral presentations, and clinician education. Outcomes were analysed using descriptive statistics and chi-square tests (significance threshold: p<0.05).

Results: A total of 154 patients were included (Phase 1: 89; Phase 2: 65). CT kidneys, ureters, and bladder (KUB) within 24 hours improved from 98% to 100%. Urgent intervention within 48 hours increased significantly (from 35% to 53%, p=0.028), while non-indicated stent placements decreased (from 83% to 57%, p=0.001). Lost stent follow-up reduced from two to 0 patients (p=0.042), and hot clinic follow-up loss dropped from 16 to one patient (p<0.001). Serum calcium testing improved (from 47% to 66%, p=0.021), and dietary counselling rates rose from 17% to 72% (p<0.001). Conservative management attempts declined (from 65% to 46%, p=0.019), with failure rates reducing from 38% to 23% (p=0.044).

Conclusion: Structured multidisciplinary interventions led to significant improvements in guideline adherence. The introduction of IT solutions, electronic recall systems, and same-day treatment pathways reduced unnecessary interventions and enhanced continuity of care. Incorporating predictive tools such as the MIMIC score into CT reporting could further optimise early stratification and decision-making.

## Introduction

Ureteric stones account for a substantial proportion of emergency urological admissions, with a lifetime prevalence of approximately 10-15% in men and 7% in women [1]. In the UK, prompt, evidence-based management is essential to minimise complications such as infection, renal impairment, or the need for avoidable surgical intervention. The National Institute for Health and Care Excellence (NICE) guideline NG118 advocates for low-dose non-contrast CT KUB (kidneys, ureters, and bladder) within 24 hours, nonsteroidal anti-inflammatory drugs (NSAIDs) as first-line analgesia, definitive treatment within 48 hours when indicated, and selective use of medical expulsive therapy (MET) for distal ureteric stones <10 mm [2]. The Getting It Right First Time (GIRFT) initiative reinforces these principles by aiming to reduce variation in care, discourage unnecessary stenting, and promote efficient outpatient pathways [3]. Despite these national recommendations, compliance varies significantly across National Health Service (NHS) trusts due to staffing pressures, logistical constraints, and inconsistent local protocols. This audit evaluated current practice in the acute management of ureteric stones at a UK district general hospital against NICE NG118 and GIRFT national guidelines and assessed the impact of targeted interventions on adherence to these standards between two audit cycles.

## Materials and methods

Design and setting 

A retrospective two-phase quality improvement audit was conducted at a district general hospital in the UK. The aim was to assess clinical practice in the acute management of ureteric stones and evaluate the impact of targeted service interventions over two audit cycles.

This audit was designed to evaluate current practice against national standards outlined (Table 1) in the NICE NG118 guideline and GIRFT recommendations for the management of ureteric stones.

**Table 1:** Summary of NICE NG118 and GIRFT recommendations for acute ureteric stone management. Adapted from the National Institute for Health and Care Excellence (NICE) guideline NG118 (NICE NG118) [2] and Getting It Right First Time (GIRFT) report [3]. CT KUB: CT kidneys, ureters, and bladder; ESWL: extracorporeal shock wave lithotripsy; MET: medical expulsive therapy; NSAIDs: nonsteroidal anti-inflammatory drugs; URS: urgent primary ureteroscopy

Recommendation Area	NICE NG118	GIRFT Priorities
Imaging	Low-dose CT KUB within 24 hours	Promote timely diagnosis to avoid delays
Analgesia	NSAIDs as first-line therapy	Reduce opioid overuse
Definitive management	URS or ESWL within 48h if indicated	Reduce variation, promote day-case treatments
MET (alpha-blocker)	Consider for distal stones <10 mm	Improve MET documentation and implementation
Stent use	Avoid unless infection or obstruction	Minimize unnecessary stenting and repeat procedures
Follow-up & recurrence	Offer dietary/metabolic advice and follow-up	Standardise recall and patient education

Statistical analysis

Descriptive statistics were calculated. Categorical variables were compared using the chi-square test or Fisher’s exact test; continuous variables were compared using unpaired t-tests. A p-value <0.05 was considered statistically significant. Data were stored and analysed using Microsoft Excel® (Microsoft® Corp., Redmond, WA).

Inclusion criteria

All consecutive patients aged ≥16 years presenting to the emergency department or acute admissions unit with a single ureteric stone ≤20 mm confirmed on CT KUB were included in both audit cycles. Cycle 1 included admissions from August to November 2023 and Cycle 2 from July to September 2024.

Exclusion criteria

We excluded patients who self-discharged, had an alternative diagnosis, were younger than 16 years, were pregnant, had prior intervention such as a stent or ESWL, or had large/complex renal stones (including staghorn calculi) or bladder stones.

Data collection

Patient data were collected retrospectively using the hospital's electronic medical records system (Cerner PowerChart). Two patient cohorts were identified for analysis: Phase 1 included admissions from August to November 2023 (n=89), and Phase 2 covered July to September 2024 (n=65). The intervention phase lasted four months (March to June 2024).

For each patient, the following data were recorded: demographics, presenting symptoms, timing of CT KUB imaging, use of NSAIDs as first-line analgesia, stone size and location, lab findings, type and timing of intervention (if applicable), stent placement, discharge documentation, follow-up arrangements, and metabolic evaluation (including serum calcium testing and dietary counselling).

Interventions implemented between phases

Following the first audit cycle, a series of structured interventions were introduced to address areas of non-compliance and improve clinical outcomes. These included the implementation of a hot (acute) day-case ESWL pathway and the development of a primary ureteroscopic stone removal pathway. Auto-text templates were created within Cerner discharge summaries to standardise the documentation of dietary advice and stent instructions. An electronic stent recall registry was also established to reduce the risk of lost follow-up.

A directive was introduced requiring clinicians to document stent placements without absolute indications and to improve audit transparency and service planning. These records will also support engagement with hospital management for potential expansion of urgent stone intervention services.

Additional educational interventions were also implemented. Posters summarising key recommendations were displayed in the department, and targeted teaching sessions were delivered to junior doctors to improve in providing dietary advice and stent-related information in discharge summaries. Meetings were held with the specialist stone nurse to improve follow-up coordination, and cases involving lost follow-up were formally discussed during departmental morbidity and mortality meetings.

The multidisciplinary hot primary URS pathway involves collaboration between urology specialty registrars, pre-assessment nursing staff, and the theatre booking team to expedite definitive treatment for appropriate patients. Finally, the Multivariable Integration of clinical, Imaging and Calculated (MIMIC) score was introduced as an educational tool to guide clinical decision-making.

## Results

Patient demographics and clinical presentation

In Phase 1, the mean age of patients was 54.2 years (range: 27-87), and 80% were male. In Phase 2, the mean age was slightly higher at 55.8 years (range: 25-93), with 66% male and 34% female patients. In both phases, approximately 68% of patients presented with flank or abdominal pain and presented to A&E within 24 hours. Associated symptoms included nausea, vomiting, dysuria, and dark-coloured urine, consistent with classical presentations of renal colic.

Diagnostic imaging and preventive measures

**Figure 1:**
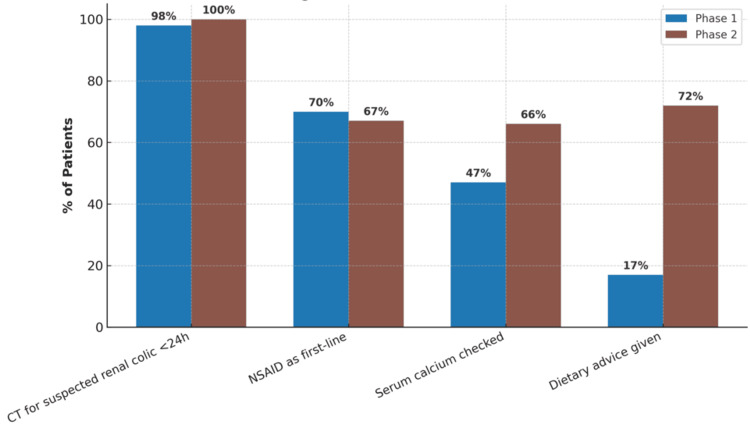
Diagnostic and preventive measures across audit cycles.

As shown in Table 2 and visualised in Figure 1, nearly all patients received appropriate imaging with a low-dose CT scan for suspected renal colic within 24 hours of presentation, with compliance improving slightly from 98% in Phase 1 to 100% in Phase 2 (p=0.31). Although this difference was not statistically significant, it reflects strong adherence to NICE NG118 recommendations for prompt imaging in suspected renal colic [2]. Use of NSAIDs as first-line analgesia remained consistent across both cycles (Phase 1: 70%; Phase 2: 67%; p=0.71), with no significant change observed.

**Table 2:** Key outcomes. ↑: Statistically significant increase; ↓: Statistically significant decrease; CT KUB: CT kidneys, ureters, and bladder; ESWL: extracorporeal shock wave lithotripsy; MET: medical expulsive therapy; NSAIDs: nonsteroidal anti-inflammatory drugs; URS: urgent primary ureteroscopy

Metric	Phase 1	Phase 2	p-value	Significance
CT KUB within 24 hours for suspected renal colic	98%	100%	0.31	Not significant
NSAIDs as first line	70%	67%	0.71	Not significant
Conservative management offered	65%	46%	0.019	Significant ↓
Conservative management failure	38%	23%	0.044	Significant ↓
Urgent intervention <48 hours	35%	53%	0.028	Significant ↑
Primary URS performed	6	7	NA	NA
Hot (acute) ESWL	1	7	<0.01	Significant ↑
Stent without sepsis	83%	57%	0.001	Highly significant ↓
Lost stent follow-up	2	0	0.042	Significant ↓
Lost to hot clinic follow-up	16	1	<0.001	Highly significant ↓
Serum calcium checked	47%	66%	0.021	Significant ↑
Dietary advice given	17%	72%	<0.001	Highly significant ↑

Notably, significant improvement was observed in two key preventive measures. Serum calcium testing increased from 47% in Phase 1 to 66% in Phase 2 (p=0.021), supporting more consistent evaluation of metabolic risk factors for stone recurrence. Additionally, dietary advice provision rose dramatically from 17% to 72% (p<0.001), following implementation of targeted interventions. These included structured junior doctor teaching sessions emphasising guideline-based dietary counselling, development of educational posters for clinical areas, and integration of auto-text templates for dietary guidance within Cerner (a widely used UK-based clinical documentation and electronic health record system). 

Treatment approaches and outcomes

**Figure 2:**
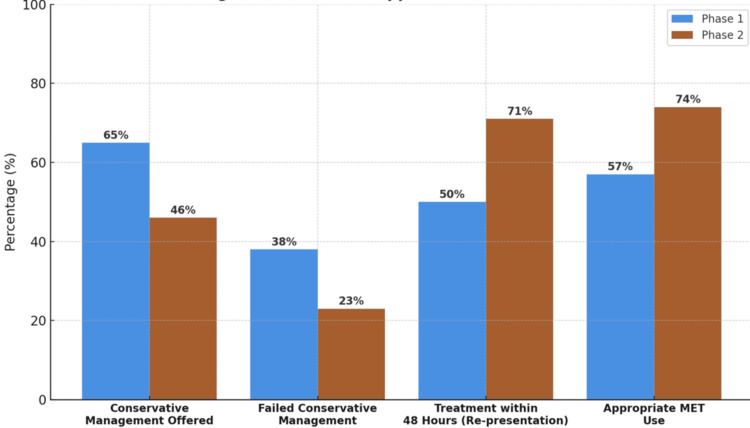
Treatment and outcomes. MET: medical expulsive therapy

There was a significant decrease in the number of patients managed conservatively, from 65% in Phase 1 to 46% in Phase 2 (p=0.019). Additionally, the failure rate of conservative management, defined as recurrence of symptoms requiring stone removal intervention, fell significantly from 38% to 23% (p=0.044). Among those who failed conservative management, 50% in Phase 1 received urgent intervention within 48 hours of re-presentation, compared to 71% in Phase 2, as demonstrated in Figure 2 and detailed in Table 2.

The use of MET also improved. In Phase 1, 43% of eligible patients did not receive MET, while this dropped to 26% in Phase 2, indicating enhanced compliance with evolving practice guidelines. This trend is visually supported by Figure 2, which illustrates the shift toward better adherence across multiple treatment-related metrics. The European Association of Urology (EAU) guidelines advocate selective MET use for well-selected patients with distal ureteric stones <10 mm, citing potential benefits in facilitating stone passage and reducing the need for intervention [4].

Together, these outcomes reflect the positive impact of systematic pathway optimisation, education, and clinical engagement in improving decision-making and treatment delivery in acute stone management.

Definitive management and surgical intervention

Urgent definitive treatment within 48 hours of initial presentation increased significantly from 35% in Phase 1 to 53% in Phase 2 (p=0.028). This improvement aligns with GIRFT recommendations to minimise delays in stone clearance, which can reduce complications and cost [3]. URS was performed in six patients in Phase 1 and seven patients in Phase 2. Additionally, hot ESWL usage increased from one to seven cases (p<0.01), as shown in Table 2.

Stent utilisation and follow-up

**Figure 3:**
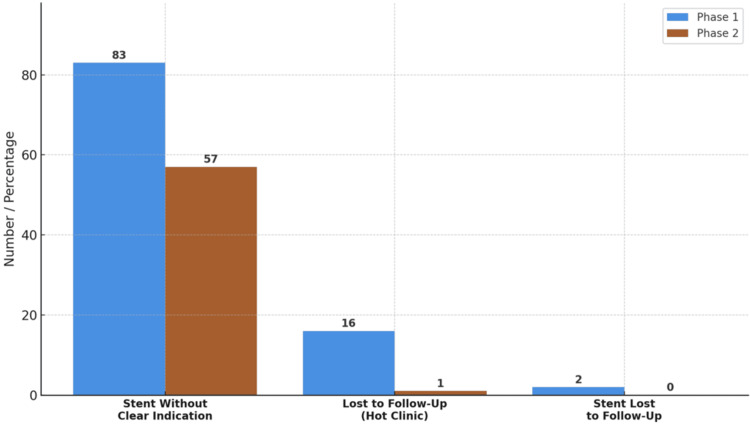
Stent usage and follow-up outcomes.

There was a statistically significant reduction in non-indicated stent placement defined as insertion without sepsis or obstruction from 83% in Phase 1 to 57% in Phase 2 (p=0.001), as seen in Figure 3. This shift highlights increased awareness of evidence-based stenting practices, consistent with GIRFT guidance discouraging the overuse of stents.

Follow-up measures also showed major improvements. Sixteen patients were lost to follow-up from the hot stone clinic in Phase 1, compared to just one patient in Phase 2 (p<0.001). Additionally, two patients with indwelling stents in Phase 1 were entirely lost to follow-up, raising critical patient safety concerns. In Phase 2, no patients with stents were lost, marking a substantial improvement in continuity of care (Figure 3, Table 2).

Following the first audit cycle, the MIMIC scores a local clinical stratification tool based on stone size, location, Hounsfield unit density, hydronephrosis, NSAID use, and prior history was encouraged to support early decision making. In Phase 2, retrospective scoring revealed that 21 patients had a score ≥6, indicating a low likelihood of spontaneous passage. Despite this, only nine of these patients (43%) received definitive treatment during their initial admission.

## Discussion

Ureteric stones remain a common cause of emergency urology admissions, with significant variation in management across healthcare systems. Adherence to national guidelines such as NICE NG118 and GIRFT has been shown to reduce variation in care, minimise unnecessary interventions, and improve outcomes [2,3]. Our two-cycle audit aimed to evaluate clinical practice in a UK district general hospital and assess the effect of structured service improvements.

This audit highlights how targeted, system-level interventions can drive meaningful improvement in the acute management of ureteric stones. Several key metrics improved across cycles, notably in urgent intervention delivery, stent governance, and preventive care, all closely aligned with NICE NG118 and GIRFT recommendations (Table 1) [2,3].

Despite high baseline compliance with CT imaging and NSAID use, Phase 2 demonstrated strengthened consistency in applying these standards (Figure 1, Table 2). This indicates a well-established foundation of early diagnostic and analgesic practice. However, a major gain was seen in preventive measures - serum calcium screening and dietary counselling both increased significantly. These improvements followed structured junior doctor education, Cerner auto-text implementation, and local poster presentation. Preventive strategies are supported by studies that link metabolic evaluation and lifestyle guidance to reduced stone recurrence [4,5]. Coe et al. and Curhan et al. both demonstrated a reduction in recurrence with dietary modification and biochemical assessment [6,7]. Such multifaceted interventions reinforce how simple educational tools can impact adherence to long-term stone prevention strategies.

The rate of conservative management dropped from 65% to 46%, with corresponding reductions in failure rates and increases in escalation to definitive treatment within 48 hours (Figure 2). These findings reflect improved risk assessment and earlier recognition of patients unlikely to benefit from non-intervention. Notably, increased use of MET suggests a more tailored approach to conservative care. Though international consensus on MET remains cautious, especially following conflicting trial evidence, recent summaries by NICE ESUOM40, EAU, and Cochrane reviews support its selective use for distal stones <10 mm [4-6,8]. The SUSPEND trial questioned MET’s role [9], but the Cochrane review by Campschroer et al. [8] highlighted potential benefits in specific subgroups. Our data suggest that enhanced MET use may have contributed to reduced failure rates and fewer delayed interventions.

While urgent definitive treatment improved (from 35% to 53%), implementation of hot URS and ESWL remained suboptimal due to ongoing logistical constraints. Staffing shortages, broken ESWL equipment, and pressure on emergency theatres were persistent barriers. Despite this, a pathway involving registrars, pre-assessment nurses, and coordinators has been established to improve theatre planning and patient selection. A third cycle will assess its full impact. These findings mirror other improvement efforts, such as the British Association of Urological Surgeons (BAUS) renal colic audit, which reported improved access to emergency stone surgery through standardised pathways [10].

This audit clearly demonstrated a reduction in non-indicated stent use (from 83% to 57%), alongside a complete elimination of patients lost to follow-up with stents in Phase 2 (Figure 3, Table 2). These improvements followed the development of an electronic stent registry with real-time data entry and monitoring. To ensure appropriate use, all urgent stent insertions were assessed for valid clinical indications - primarily sepsis. White cell count, C-reactive protein (CRP), and documented fever were reviewed to rule out infection. Urological guidelines support decompression in cases of infected obstructed systems to prevent urosepsis [11].

Forgotten stents remain a medicolegal and clinical risk, with known complications including infection, encrustation, obstruction, and even malignancy in chronic cases [6,12]. A study by Lam et al. showed complication rates as high as 11% in forgotten stents [13]. The implemented safety measures - clinician prompts, nurse-led coordination, and automated reminders - serve as a model for other centres aiming to mitigate stent-related complications in a resource-constrained environment.

The implementation of a locally developed risk stratification tool (MIMIC score - Table 3) served as an exploratory aid to support early decision‑making. Although not a validated or peer‑reviewed tool, it incorporates established predictors of stone passage such as stone size, location, Hounsfield unit density, presence of hydronephrosis, NSAID use, and history of previous stones [6].

**Table 3:** MIMIC score - adapted from clinical criteria consistent with predictors of spontaneous passage. Multivariable Integration of clinical, Imaging and Calculated (MIMIC) score - adapted from clinical criteria consistent with predictors of spontaneous passage described in [6]. Although the MIMIC score itself is not published in a PubMed-indexed journal, its parameters are consistent with those established in published literature. NSAID: nonsteroidal anti-inflammatory drugs

MIMIC Score Parameters	Points
Stone size ≥6 mm	2
Proximal stone location	2
Hounsfield units >1000	1
Moderate/severe hydronephrosis	1
No NSAID administration	1
Previous stone history	1

Financially, unnecessary stent use and repeat procedures are unsustainable. NICE estimates the average cost of ureteroscopy at ~£1,500, with added stenting procedures exceeding £1,000 each [2]. Reducing just 30 unnecessary stents per year could save £30,000-£40,000, excluding indirect costs from delayed treatment, patient morbidity, and outpatient burden [6]. Interventions such as stent audit trails and early escalation pathways have potential for cost-neutral implementation with high return on patient safety and throughput.

Our results align with other national improvement programmes. Finch et al. reported better access to emergency intervention and standardisation of care following pathway implementation across multiple sites [10]. Johnston et al. further highlighted the economic burden of repeat interventions and the importance of getting it right the first time [14].

This audit has several limitations. Its retrospective, single‑centre design and modest sample size may limit the generalisability of the findings to other settings. The accuracy of the results is dependent on the completeness of electronic medical records, introducing potential data capture bias. Selection bias may also be present despite the inclusion of all consecutive eligible patients, and a possible Hawthorne effect cannot be excluded, as staff awareness of the audit may have influenced behaviour during the second cycle. However, its strength lies in the real-world applicability of its findings and practical interventions that can be adapted in other NHS trusts.

In conclusion, this audit demonstrates that multidisciplinary collaboration, information technology (IT) integration, and structured clinical pathways can drive measurable improvements in guideline adherence, patient safety, and service efficiency in acute ureteric stone care.

## Conclusions

This two-cycle audit highlights the positive impact of structured adherence to NICE NG118 and GIRFT recommendations in the management of acute ureteric stones. Key improvements included a reduction in unnecessary stent insertions, better documentation and implementation of MET, earlier access to definitive treatments through the introduction of a day-case ESWL pathway, and a significant decrease in failed conservative management rates. Importantly, multidisciplinary collaboration and IT integration enhanced follow-up systems, eliminating missed stent recalls and reducing hot clinic attrition. These outcomes demonstrate that protocol-driven, team-based approaches can meaningfully improve patient safety, streamline care, and align practice with national standards. Ongoing monitoring and reinforcement of these strategies will be essential to sustain and build upon these gains.

## References

[1] Khan SR, Pearle MS, Robertson WG (2016). Kidney stones. Nat Rev Dis Primers.

[2] National Institute for Health and Care Excellence (NICE) (2019). NICE guideline - renal and ureteric stones: assessment and management. BJU Int.

[3] John JB, Gray WK, O'Flynn K, Briggs TW, McGrath JS (2024). The getting it right first time (GIRFT) programme in urology; rationale and methodology. BJU Int.

[4] Skolarikos A, Jung H, Neisius A (2025). EAU Guidelines on Urolithiasis. Biophys Chem.

[5] Assimos D, Krambeck A, Miller NL (2016). Surgical management of stones: AUA/Endourological Society guideline, part I. J Urol.

[6] Coe FL, Evan AP, Worcester EM, Lingeman JE (2005). Kidney stone disease. J Clin Invest.

[7] Curhan GC, Willett WC, Speizer FE, Spiegelman D, Stampfer MJ (1997). Comparison of dietary calcium with supplemental calcium and other nutrients as factors affecting the risk for kidney stones in women. Ann Intern Med.

[8] Campschroer T, Zhu X, Vernooij RW, Lock MT (2018). Alpha-blockers as medical expulsive therapy for ureteral stones. Cochrane Database Syst Rev.

[9] Pickard R, Starr K, MacLennan G (2015). Medical expulsive therapy in adults with ureteric colic: a multicentre, randomised, placebo-controlled trial. Lancet.

[10] Finch W, Calvert RC, Fowler S, Hermans L, Dickinson A, Smith D (2023). Enabling national improvement in quality of care for renal colic. BJU Int.

[11] Wolf JS Jr, Bennett CJ, Dmochowski RR, Hollenbeck BK, Pearle MS, Schaeffer AJ (2008). Best practice policy statement on urologic surgery antimicrobial prophylaxis. J Urol.

[12] Robert M, Boularan AM, El Sandid M, Grasset D (1997). Double-J ureteric stent encrustations: clinical study on crystal formation on polyurethane stents. Urol Int.

[13] Lam JS, Gupta M (2004). Update on ureteral stents. Infect Urol.

[14] Johnston SS, Chen BP, Rai P (2022). Incremental healthcare cost implications of retreatment following ureteroscopy or percutaneous nephrolithotomy for upper urinary tract stones: a population-based study of commercially-insured US adults. Med Devices (Auckl).

